# A Nano-Thin Film-Based Prototype QCM Sensor Array for Monitoring Human Breath and Respiratory Patterns

**DOI:** 10.3390/s150818834

**Published:** 2015-07-31

**Authors:** Roman Selyanchyn, Shunichi Wakamatsu, Kenshi Hayashi, Seung-Woo Lee

**Affiliations:** 1 Graduate School of Environmental Engineering, The University of Kitakyushu, 1-1 Hibikino, Wakamatsu, Kitakyushu 808-0135, Japan; E-Mail: romanselyanchyn@gmail.com; 2 WPI International Institute for Carbon-Neutral Energy Research (WPI-I^2^CNER), Kyushu University, 744, Motooka, Fukuoka 819-0395, Japan; 3 Chitose Technical Center, Nihon Dempa Kogyo Co. Ltd., 1-3-1, Minami-Chitose, Hokkaido 066-0009, Japan; E-Mail: wakamatu@ndk.com; 4 Graduate School of Information Science and Electrical Engineering, Kyushu University, 744 Motooka, Fukuoka 819-0395, Japan; E-Mail: hayashi@ed.kyushu-u.ac.jp

**Keywords:** quartz crystal microbalance sensor, nano-thin film, respiratory measurement, lung function test

## Abstract

Quartz crystal microbalance (QCM) sensor array was developed for multi-purpose human respiration assessment. The sensor system was designed to provide feedback for human respiration. Thorough optimization of measurement conditions: air flow, temperature in the QCM chamber, frequency measurement rate, and electrode position regarding to the gas flow—was performed. As shown, acquisition of respiratory parameters (rate and respiratory pattern) could be achieved even with a single electrode used in the system. The prototype system contains eight available QCM channels that can be potentially used for selective responses to certain breath chemicals. At present, the prototype machine is ready for the assessment of respiratory functions in larger populations in order to gain statistical validation. To the best of our knowledge, the developed prototype is the only respiratory assessment system based on surface modified QCM sensors.

## 1. Introduction

The European Respiratory Society (ERS) reports that six million hospital admissions per year are due to respiratory diseases, among which 600,000 people die yearly in the EU [1]. The quantitative evaluation of human respiration and lung functions is an important task in patient objective assessment. In particular, respiratory parameters are required for the proper management of both lung obstructive (e.g., chronic obstructive pulmonary disease (COPD), asthma) and lung restrictive diseases. Lung function tests also provide useful information for diagnosis, monitoring treatment, and evaluation of the prognosis of other diseases [2]. One class of devices has been developed to provide mechanical parameters of respiration (respiratory rate, tidal volume, lung capacity, peak expiratory flow, *etc.*), while the other is used to acquire chemical breath composition (e.g., capnography for breath carbon dioxide measurement) [3]. A majority of the commercial devices for respiratory self-assessment provide only a single parameter (e.g., respiratory rate). Multi-purpose but highly sophisticated and expensive systems are available in hospitals, which can provide a rich spectrum of information about human breath and respiration. Certainly, these devices need a qualified operator to run the tests, and a physician to be able to recognize the pathology based on the respiratory test results.

Quartz crystal microbalance (QCM) is a widely used mass-sensitive transducer due to the simplicity of its operation. In most cases, QCM oscillation frequency response to the mass loaded on the active electrode surface follows the Sauerbrey Equation (1) [4]:

(1) $$\Delta f=-\frac{2f_{0}^{2}}{A\sqrt{{\text{μ}}_{q}{\text{ρ}}_{q}}}\Delta m$$

where *f_0_* is the resonance frequency of the unloaded QCM, *A* is the active area of the crystal, μ*_q_* and ρ*_q_* are the quartz shear modulus (2.947 g·cm^−1^·s^−2^) and density (2.648 g·cm^−3^), respectively. QCM-based sensors are gaining popularity in recent years [5,6] and have been used in a variety of fields, including biotechnology [7], drug and surfactant research [8,9], biosensing [10,11,12,13,14], or gas sensing purposes [15,16,17], as an element of the electronic tongue or electronic nose devices [18,19]. Recently, our group has reported the creation of reliable humidity sensors, based on QCM electrodes modified with porphyrin-based nano-assembled thin films [20], which can be also applied to daily needs, for instance, monitoring of indoor environmental quality [21].

Recently, we reported a respiration assessment system, based on QCM electrodes, possessing a surface modified with nano-assembled thin films [22]. The online breathing record (respiratory pattern) and respiratory rate were obtained using a home-made experimental set-up. As suggested in our previous work [22], a respiratory signal was obtained due to the quick response of the QCM sensors to the fast changes of relative humidity in human breath samples, depending on human breathing styles, such as speed, intensity, and time of inhalation and exhalation. In general, humidity is considered an important factor, in particular for the development of chemical sensors that are supposed to work with real (often highly humid) samples. When selective detection of a chemical is required, humidity becomes the most interfering factor. Thus, technological improvements are desired to avoid the influence of humidity. Our previous work, however, could be conducted under compromised humidity levels that were not very high and enabled respiration signal measurement. As we demonstrated, respiratory monitoring (respiratory rate and respiratory pattern) was possible with the use of surface functionalized QCM sensors. The mechanism of QCM response to human respiration was found to be related to the quick response to the humidity in human breath. Therefore, online measuring of human respiratory parameters was achieved. It was also suggested that a differentiated QCM signal has the potential to extract, not only a simple respiratory rate value (number of oscillations per second) but also other important clinical data, such as tidal and inspiratory volumes and peak inspiratory and expiratory flows [2].

The current report describes the progress of the development of a prototype device for respiration/breath assessment, based on QCM mass-sensitive sensors, modified with the nano-assembled thin films [16,20] introduced in our previous work [22]. The proposed system, with all necessary built-in units, has been designed for breath-analyzing purposes, including the assessment of mechanical parameters and the chemical composition of breath.

## 2. Experimental Section

### 2.1. Materials

Poly(diallyldimethylammonium chloride) (PDDA, M_w_ = 200,000–350,000 g·mol^−1^, 20 wt% in H_2_O), poly(allylamine hydrochloride) (PAH, M_w_ = 58,000 g·mol^−1^), and β-cyclodextrin, sulfated sodium salt (β-SCD, M_w_ = 1134.98 g·mol^−1^) were purchased from Sigma-Aldrich, St Louis, MO, USA. Tetrakis(4-sulfophenyl)porphine (TSPP, M_w_ = 934.99 g·mol^−1^), tetrakis(4-carboxyphenyl)porphyrin (TCPP, M_w_ = 790.79 g·mol^−1^), and 4-Sulfocalix[8]arene (SCA[8], M_w_ = 1489.45 g·mol^−1^) were purchased from Tokyo Kasei, Japan. An aqueous solution of silica nanoparticles (SiO_2_ NPs, SNOWTEX-20L, particle size 40–50 nm) was purchased from Nissan Chemical Industries, Ltd. (Tokyo, Janpan). Other chemicals used as solvents were of analytical grade purity and obtained from commercial sources. All of the used chemicals were guaranteed reagents, and used without further purification. Deionized pure water (18.3 M·Ω·cm) was obtained by reverse osmosis, followed by ion exchange and filtration (Millipore, Direct-QTM).

### 2.2. Sensor Fabrication

**Figure 1:**
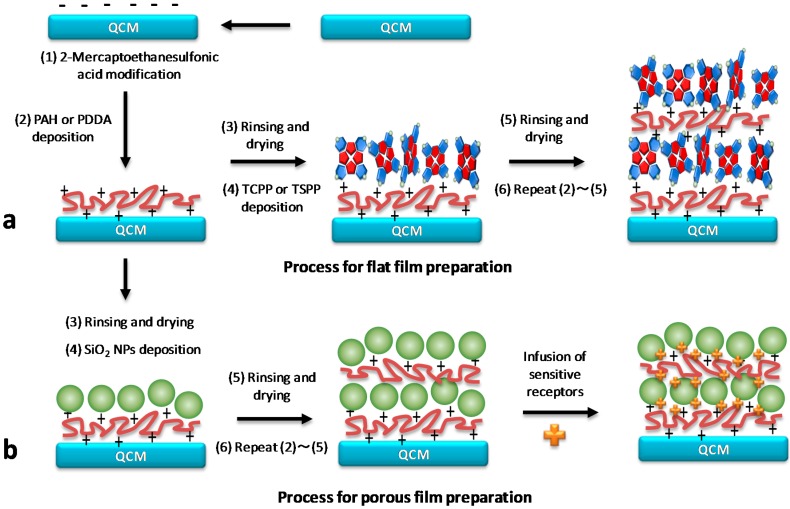
General illustration concept of the QCM surface modification used in the current work. (**a**) Deposition of flat film on the QCM surface; (**b**) formation of porous film on the surface of the QCM electrode, followed by infusion of a sensitive molecular receptor (modification of selectivity).

Gold-coated AT-cut QCM electrodes with an initial resonant frequency of 9 MHz were used in this study, provided by Nihon Dempa Kogyo (NDK) Ltd., Japan. The electrode surface was modified using layer-by-layer (LbL) alternate adsorption of oppositely charged polyions, similar to the previously reported methods [20,22,23,24]. Shortly, after cleaning and drying, QCM electrodes were treated overnight in a 2-mercaptoethanesulfonic acid solution (10 mM in ethanol) in order to obtain a negatively charged surface, and then 10 layer films were deposited. To prepare functional flat films, 10 cycles of alternate adsorption of polycation (PAH or PDDA) and anionic compound (TSPP or TCPP) onto the quartz crystal were undertaken (one cycle consist of a cationic/anionic bilayer) (Figure 1a). In every case, the alternate film had the porphyrin compound as the outermost layer.

For deposition of porous film, the approach described in [23] was used. It is similar to the flat film formation approach, where negatively charged SiO_2_ NPs are used as an anionic component. After the first layer PAH deposition, washing with water, and drying under N_2_ gas, the QCM electrode was immersed into a solution of SiO_2_ NPs for 15 min at 25 °C. The procedure was repeated until 10 cycle coatings (where one cycle is a SiO_2_/PAH bilayer) were prepared by the alternate adsorption of PAH and SiO_2_ NPs onto the QCM. Then, the fabricated films were infused with a sensitive receptor according to the following procedure. The QCM electrode coated with (SiO_2_/PAH)_10_ was immersed into an 1 mM aqueous solution of TSPP, β-SCD or SCA[8] for 30 min at 25 °C. After the infusion of compound into the (SiO_2_/PAH)_n_ film, the QCM electrode was rinsed with deionized water and dried using N_2_ gas. The self-assembly and infusion processes were monitored by measuring the QCM resonance frequency after every deposition cycle or infusion process.

Electrodes with six different films, used for respiratory recording optimization, are given in Table 1. We intentionally selected three electrodes with flat films (10 layers, thickness ~20–30 nm) and three porous films with infused chemical receptors (10 layers, thickness ~500 nm) to investigate how the coating morphology and chemical nature can influence the responses of the machine to human respiration. The thicknesses of the porous films can be estimated from the QCM frequency shifts or scanning electron microscopy (SEM) observation [23]. However, SEM resolution is not sufficient to measure the thickness of flat film; therefore, the QCM frequency shift due to film deposition (Δ*F^film^*) is used as an estimate of the deposited mass. Subsequently, the thickness can be calculated using the bulk densities of the materials used for film fabrication. Atomic force microscopy (AFM) can be also used if the information about the thickness is critical, as we reported elsewhere [20,25]. In this work we did not measure the thickness, and only the Δ*F^film^* values, measured by QCM, were used to calculate the deposited mass, also given in Table 1. The mass of the deposited films (*m^film^*) and infused compounds (*m^inf^*) were calculated using the Sauerbrey Equation (1). Considering the parameters of the QCM electrode used in this study (fundamental frequency and active electrode surface area), a Δ*F* of 1 Hz corresponds to a Δ*m* of *ca*. 1.07 ng.

**Table 1:** Electrodes used in the experimental tests.

QCM Number *	Surface Modification	–Δ*F^film^*, Hz	*m^film^*, µg	Infused Compound	–Δ*F^inf^*, Hz	*m^inf^*, µg
F1	[PAH/TCPP]_10_	–1242	1.33	*Not applicable*		
F2	[PDDA/TCPP]_10_	−1017	1.09			
F3	[PDDA/TSPP]_10_	−891	0.95			
P1	[PAH/SiO_2_]_10_	−19,240 ± 120	20.59 ± 0.13	SCA[8]	−1366	1.46
P2	[PAH/SiO_2_]_10_			β-SCD	−336	0.36
P3	[PAH/SiO_2_]_10_			TSPP	−1719	1.84

* Electrode conditional number: F and P stand for “flat” and “porous”, respectively.

### 2.3. Measurement Setup

**Figure 2:**
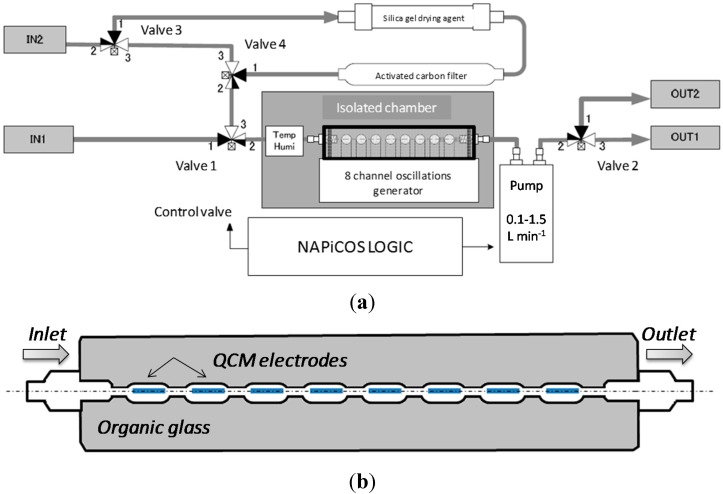
(**a**) Block-scheme of the prototype QCM sensor array; (**b**) Top-view of the geometry of QCM electrodes containing chamber, supporting the proper flow through all 8 electrodes independent on position in respect to flow.

A brand new QCM prototype machine was built by NDK Ltd. (Tokyo, Japan) [26], based on the results of our previous work [22]. The developed system included new features that were not possible in the previously used, home-made version, namely chamber temperature control, embedded reference temperature and humidity sensors, automatic flow control, and sample introduction and possibility to acquire QCM frequencies up to 16 times per second. A schematic overview of the prototype sensor system fabricated by NDK Ltd. is shown in Figure 2a. The machine has two inlets: one for sample gas introduction and the other for reference gas flow. Operation cycles of the sample introduction and system cleaning are controlled by automatic valves 1–4. For cleaning purposes, the sensor system is flushed by the air that is cleaned and dried through the activated carbon and silica gel, respectively. Delivery of the sample and reference gases is done by the use of a Sibata^®^ mini-pump connected to the QCM chamber outlet. Temperature and humidity inside the chamber are recorded by additionally installed reference sensors, which are located in an isolated chamber in proximity to the QCM array. Additionally, the chamber is designed to control the inside environment temperature in the range of 20 to 40 °C. The temperature and RH sensors are, respectively, SII S-5814A (accuracy ±2.5 °C, working range −30 °C to +100 °C) and TDK CHS-UPS (accuracy ±3%, measurement range 5% to 95% RH). Device operation is controlled by software (NAPiCOS 30A Real Time Monitor v.1.0) also developed by NDK Ltd. [26].

System optimization parameters include the values that can be controlled by the device settings. In the current prototype sensor system, these parameters are: flow rate of the sample gas, temperature inside the chamber, frequency sampling rate, and electrode position inside the chamber. In this study, all these parameters were optimized by assessing the responses to human respiration and the saturated humidified air as a model of human exhaled breath.

An important feature of the eight-electrode system is the chamber containing the electrodes. Several types of chambers were tested, and the best performance was obtained in the chamber that is believed to support optimal and laminar flow of the gas sample through the electrodes. Schematically, the chamber geometry is shown in Figure 2b. It has linear symmetrical geometry and is designed to keep the total volume small, therefore diminishing the gradient of temperature and humidity along the chamber.

### 2.4. Experimental Conditions

Humidified air in this work was prepared by flowing ambient air through a glass bottle saturated with water vapor. In order to compare the sensitivity of the different electrodes to humidity, the sensor array was supplied by the saturated RH air samples taken from the headspace, and responses were compared to different sensors. The period needed for reaching 90% of maximum response was used to estimate the response time of the sensor. All respiration measurements were performed with the same person breathing, in order to be able to confirm the different electrodes’ responses to the same sample. Breathing protocol was established in our previous work [22], including normal tidal breathing and 5 min of measurement used for optimization tests.

## 3. Results and Discussion

### 3.1. Response of the Prototype System to Human Respiration

**Figure 3:**
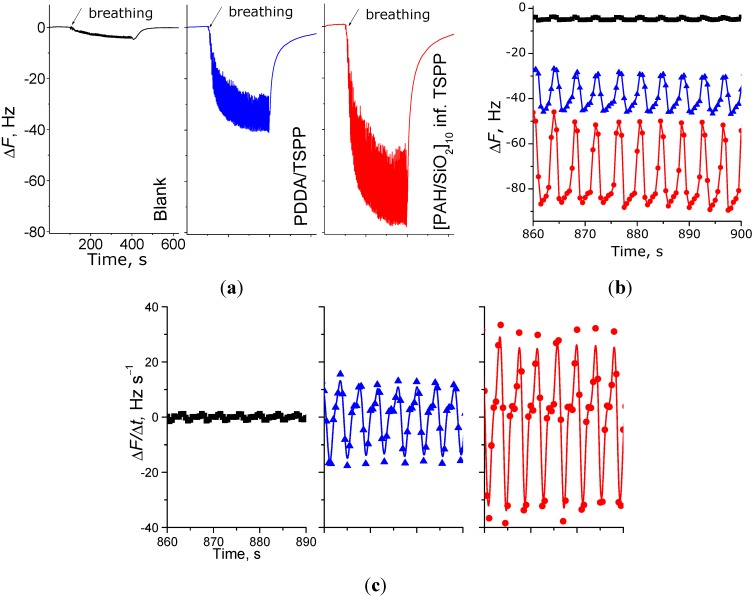
Response of the three different electrodes (blank, coated with PDDA/TSPP (F3) flat film and [PAH/SiO_2_]_10_ inf. TSPP (P3) porous film) to the breathing, demonstrating the influence of the coating. (**a**) General view of frequency shift behavior during the 5 min respiration test; (**b**) magnified view of the frequency oscillation at saturation point; (**c**) first derivative of frequency (speed of frequency change) at saturation point.

As has been shown in previous work [22], the surface modification of the electrode is crucial to obtain good responses to respiration signals. In the current study, we have extended the number of different coatings, as given in Table 1. Figure 3 demonstrates the effect of the coating on the response of human respiration, and it is seen that electrodes with coatings (flat PDDA/TSPP film and [PAH/SiO_2_]_10_ infused with TSPP porous film) are far more responsive than unmodified electrodes (indicated as blank in Figure 3).

### 3.2. Temperature Influence on Sensors Performance

**Figure 4:**
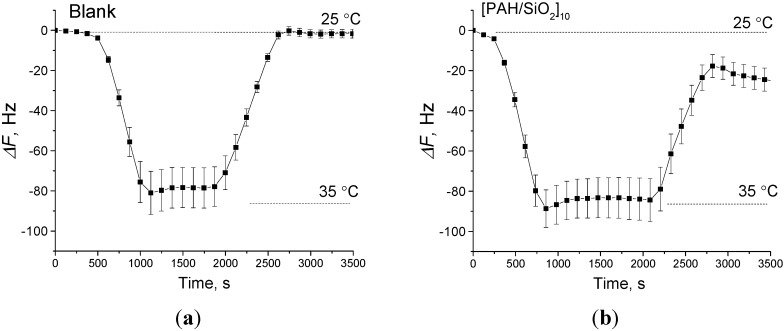
Response of the different QCM electrodes to the temperature change from 25 °C to 35 °C and back. Averaged frequency response for (**a**) 5 unmodified QCM electrodes; and (**b**) 5 QCM electrodes modified with a coating of [PAH/SiO_2_]_10_, respectively.

Temperature inside the chamber is one of parameters that can be easily changed in the newly developed system, using the software controlling the device. QCM responses at different temperatures at first were confirmed with two similar prototype systems installed with blank electrodes, and have shown a considerable dependence (Figure 4a), *i.e.*, decrease of fundamental frequency with the increase of temperature. This is a well-known phenomenon related to changes in the physical properties of quartz (e.g., density, Young modulus) and should be taken into account when significant temperature changes occur in parallel with test sample introduction.

Temperature influence tests were also performed for the electrodes coated with different films. The behavior of the electrodes was very similar to that shown by the blank electrodes and only small influence of deposited films was observed, namely slightly higher temperature-induced frequency changes occurred. Figure 4b shows the averaged response for five electrodes coated with porous [PAH/SiO_2_]_10_ films, demonstrating an insignificantly higher frequency decrease when heated from 25 °C to 35 °C. Therefore, the similar responses to the temperature of electrodes with deposited films indicate that the intrinsic properties of quartz are the main reason for the observed changes. For the current application, however, the physical influence of temperature on the fundamental frequency should be excluded as a possible interference. In order to avoid the interference during the assessment of samples, the measurement temperature (inside the electrode chamber) was maintained at a constant level.

### 3.3. Temperature Influence on the Respiratory Signal

**Figure 5:**
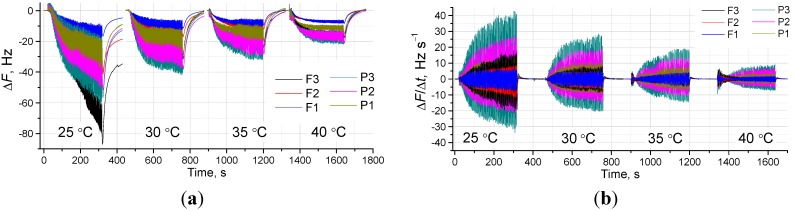

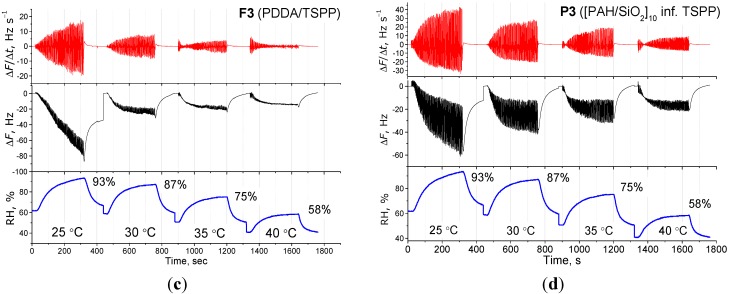
Influence of the measurement temperature on the QCM response on respiration. (**a**) Frequency shifts; and (**b**) their respective derivatives on 6 different types of films; and (**c**) typical flat-type film response ([PDDA/TSPP]_10_ as example); and (**d**) typical porous film response, ([PAH/SiO_2_]_10_ infused with TSPP as example).

In order to establish the optimized conditions for respiratory signal acquisition, the influence of temperature on the QCM breathing response has also been investigated. The breath signal at different temperatures shows that the amplitude of oscillations is strongly dependent on the chamber condition. Both frequency shifts, amplitude of frequency oscillations and derivative signals, depend on the temperature in a way that all these signals are largest at the lowest tested temperature of 25 °C, decreasing with the increase in temperature, as shown in Figure 5, for the electrodes with different functional coatings. In addition, it can be seen that the signal is coating-specific, *i.e.*, Δ*F*, amplitude of oscillations, and saturation time differ, depending on the type of deposited film.

However to explain such behavior, RH changes related to the temperature increase should be also considered, which are given in Figure 5c and d for two different types of films. It is clear that with the increase of temperature in the chamber, the relative humidity will decrease. This, in turn, will result in decreased sensor responses at higher working temperatures analogically, as happens when humidified air is supplied to the chamber (data not shown). Thus, we can conclude that the static RH change due to the introduction of a breath sample from the baseline is a major influence on Δ*F*, while the dynamical RH change during the inhalation/exhalation becomes a factor defining the oscillations of F as a response to lung movements or respiration.

**Figure 6:**
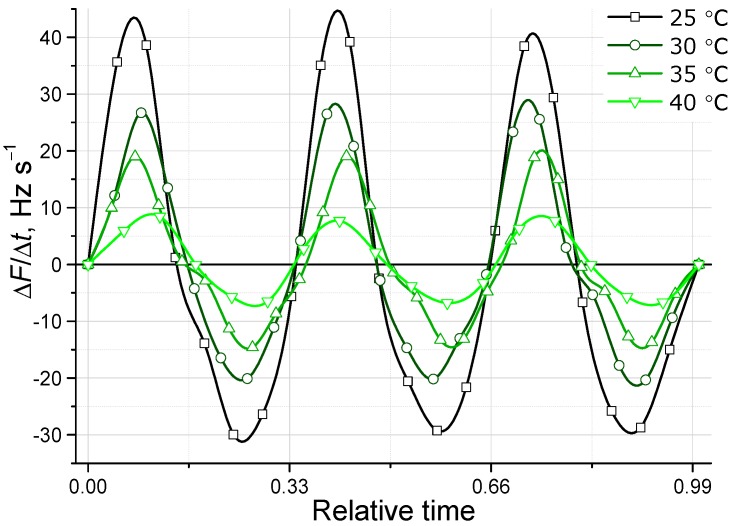
Comparison of the respiration response at different temperatures in case of the [PAH/SiO_2_]_10_ electrode infused with TSPP (F3).

Figure 6 shows the detailed comparison of three respiratory movements of the QCM response on the same electrode at four different temperatures. There are no large differences in the shape of the signal (respiratory pattern), thus, any of tested temperatures are suitable for respiratory signal assessment. However, at the lowest temperature, we have the problem of slow response for certain films (e.g., TSPP/PDDA at 25 °C in Figure 5c). Another factor we should avoid is the nonlinear response to RH that tends to appear at an RH higher than 90% [20]. Exhaled human breath appears to be *ca*. 95% humid at body temperature (35–37 °C). One place where the reduction of breath RH happens is at the mask used to collect the sample, where we have a certain mixing of exhaled breath with ambient air. Another necessary condition is to maintain the working temperature at 35 °C. This will allow all measurements to be in a very suitable RH range, where the sensors show a perfect linear response to humidity [20]. Thus, we concluded that a temperature of 35 °C in the sensing chamber is optimal for breath/respiration tests, and will be sufficient to prevent non-linear responses of the sensors.

The current study focuses only on the optimal conditions of the sensor operation. Based on the results of the influence of temperature, it can be seen that the interaction of water molecules on the electrode surface changes in terms of equilibrium. A faster response with the increase of temperature is explained by increased mobility of breath-originated gases (mainly water). Operation at higher temperatures will also be advantageous in terms of recovery to the baseline after possible adsorption of breath chemicals that are not as mobile as water and need a longer recovery time. On the other hand, a higher temperature of 40 °C leads to a much smaller response in terms of oscillation intensity, indicating that lower temperatures are more suitable for respiratory assessments.

### 3.4. Investigation of Sample Flow-Rate and Electrode Position Inside the Chamber

**Figure 7:**
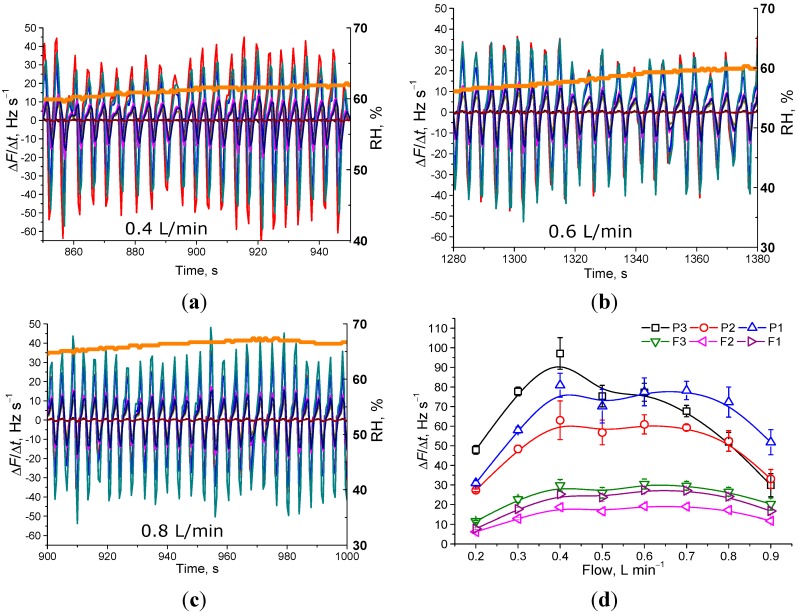
Response of the QCM sensor array to the respiration at different flows of the sample through the prototype system. (**a**–**c**) Speed of frequency change of all electrodes at three different flows (indicated in graph); (**d**) dependence of the amplitude of derivative change on the flow for all different electrodes.

In our previous work [22], it was shown that the flow of the sample through the system is also among the essential parameters for obtaining a stable and intensive response to the respiration. In the current prototype system, the pump can support a flow between 0.1 and 0.9 L·min^–1^. Figure 7a–c shows the influence of the flow on the derivative of the frequency for three particular flows 0.4, 0.6 and 0.8 L·min^–1^ and a summary of the oscillation intensities are shown in Figure 7d. As can be seen, at all flows, the response of the QCMs coated with porous films is higher than that coated with flat films. The highest signals intensity was observed at a flow of between 0.4–0.7 L·min^–1^, thus, maintaining the flow in this range is essential for optimal response

One of the essential concerns regarding the reliability of the work of the QCM sensors in the arrays is the stability of the response in regard to the position of the electrode with respect to flow in the chamber. Position can be an especially influential factor in chambers with a linear geometry, as in our case, where the electrode in oscillation channel 1 is interacting with the sample first, channel 2, second, *etc*. To check what the influence on oscillation intensity will be, we have changed the positions of electrodes with respect to flow by shifting each electrode to a different position. Indeed, we observed a small decrease in the response if the electrode is farther from the inlet. Such behavior is expected and can be explained by the several factors, mainly disturbance of flow on the end of the chamber. Nevertheless, even after the position was changed, three QCM electrodes with porous film coatings retained their greater response to the respirations in comparison with the flat film-modified electrodes.

### 3.5. Extraction of Chemical Features from Respiratory Patters

**Figure 8:**
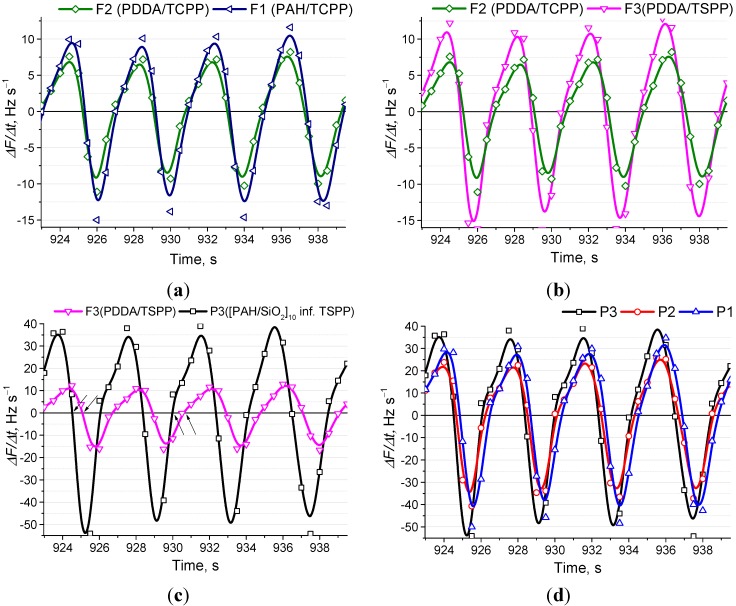
Comparison of the different QCM sensors responses to respiration. (**a**) Influence of the polycation in the PAH/TCPP and PDDA/TCPP coatings; (**b**) Influence of the anionic compound in flat film PDDA/TCPP and PDDA/TSPP coatings; (**c**) Influence of the morphology, flat *vs*. porous coating with the same sensitive receptor: PAH/TSPP compared to TSPP-infused (PAH/SiO_2_)_10_ coating; (**d**) Influence of the sensitive receptor in the same type porous (PAH/SiO_2_)_10_ coating.

In the current work, we have intentionally used different types of sensor coatings in order to estimate the potential of the system for the detection of chemical compounds in human breath. Taking into the account used for coatings we can compare the electrodes from the following points of view: influence of polycation and anionic species in flat films, influence of morphology between flat and porous films, and influence of chemical receptors introduced in the SiO_2_ NPs-based porous coating. Data representing all four of these features is given in Figure 8a–d. We can see that there is not much dependence on the polycation (Figure 8a), and both PDDA/TCPP and PAH/TCPP demonstrating similar responses. The slightly higher response of the PAH/TCPP film may be caused by its higher total frequency shift after film deposition (see Table 1). When the different porphyrins with the same polymer are compared, the difference becomes more significant in the PDDA/TSPP film (Figure 8b), whereas its total frequency shift after film deposition is smaller than that of the PDDA/TCPP film (see Table 1). This suggests that the TSPP demonstrates a higher response to human breathing. It is known that the TSPP represents a highly sensitive compound to gaseous ammonia, as we have demonstrated previously [21,22], and the LbL coating of PAH/TSPP is also highly responsive to relative humidity [20,24]. It is possible that the combination of such features results in the higher response of the PAH/TSPP sensor to breathing, compared to the PAH/TCPP, as it is known that, in addition to the high humidity, human breath also contains a reasonable amount of gaseous ammonia, reaching up to a few ppm in normal conditions.

When the influence of morphology is compared for the coatings with the same sensitive receptor (TSPP), we see even greater differences. The amplitude of response is much higher in the case of an electrode with a porous coating, as shown in Figure 8c. Moreover, the speed of response of a sensor with a porous coating is higher, as shown by arrows in the same figure. The observed result is also expected, owing to obviously higher diffusion rates in the porous coating compared to the flat one. Not only is faster access of the gas to the sensitive centers contributing to the higher response, but also the higher content of the sensitive compound itself that can be seen when the coatings are compared (see Table 1). The content of the TSPP, infused into the porous coating, is larger than the whole flat film coating itself; thus, this factor is most likely the main contributor to the observed higher response.

Finally, we can compare the influence of sensitive receptors introduced in the porous coating. All compounds are widely used in various types of sensors. Cyclodextrins (CDs) are especially promising for sensor fabrication. In most cases, CDs are used in combination with other components [27] because they can serve as macrocyclic hosts. Similarly, calixarenes are also derivatized or complexed with other molecules in order to build a chemical sensor [28,29]. In our case, we can see that all three electrodes modified with porous coatings have shown greater responses than the flat films; however, there is not much of a significant difference between them. The TSPP-infused electrode showed the highest response and it can be explained by its known sensitivity to humidity and ammonia. Human breath has an extremely complex matrix of chemicals at very low concentrations [30]. However, we believe that the electrodes used in our system could be selected in a way that it will be possible to detect at least some important and simple analytes, for instance, potential disease biomarkers, such as ammonia, acetone, or isoprene.

### 3.6. Dependence of the Signal on the Sampling Rate

The other important parameter of respiratory signal acquisition by the developed QCM system is the rate of frequency sampling, as it defines the precision of the recorded signal. The frequency sampling rate is limited by the electronic circuit of the machine. A commercial analog eight-channel Napicos system has a frequency-sampling rate fixed to one frequency per second for each of the eight channels. The maximum possible sampling rate, of 16 frequencies per second, was realized in the present prototype, which is a total sample possible for all eight channels. Namely, when all eight electrodes are installed, each channel can record two frequencies per second, giving a total number of 16 measurements. Respectively, four electrodes can be measured four times per second, for two electrodes, eight times, and, finally, for one electrode 16 times.

**Figure 9:**
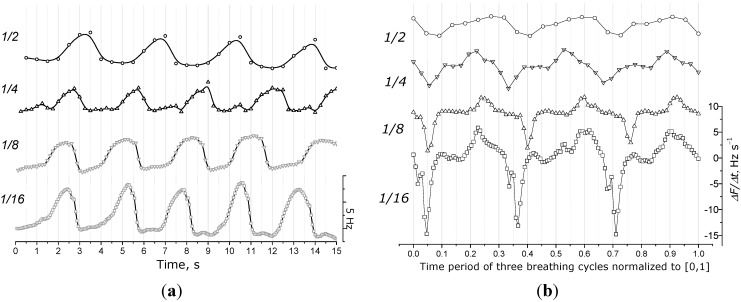
QCM frequency shifts and their derivatives in response to human respiration obtained at different sampling rates (◯ *1/2*, △ *1/4*, ▽ *1/8* and ☐ *1/16*). (**a**) Frequency shift response; (**b**) respective derivative signal (normalization performed to compare three breathing signals with different respiratory rates).

Human respiration is a quickly changing physicochemical process with a full breathing cycle (inhale and exhale) normally taking 3–5 s [2]. The results of breathing signals *F = f_1_*(*t*) and *dF/dt = f_2_*(*t*) of the same person, at different sampling rates, are given in Figure 9. By increasing the sampling rate, both QCM frequency and its derivative signal are changed considerably. In cases of the low sampling rates of *1/1* and *1/2* (here and further for sampling rate notation *1/k* is used, where *k* means the number of the sampling frequency per second), the respiratory signal is represented by a line similar to an ordinary sinusoid. However, when higher sampling rates are applied, we can observe the signals more precisely, reflecting the human breathing process with a short hold when exhaling is completed, and with a sharp change of frequency when inhaling is changed by exhaling (Figure 9a). The occurred change can be seen more clearly in the case of the frequency derivative signal that reflects the speed of the processes taking place at the QCM interface. Based on the data given in Figure 9b, we conclude that sampling rates of *1/2* and *1/4* are quite imprecise to assess respiration signals, however, still enough to count respiratory rate. In order to provide accurate measurement of other important respiratory parameters (e.g., expiratory flow measurement), higher sampling rates (at least *1/8*) have to be applied. A sampling rate of *1/16* is, thus, the best option for precise, detailed, dynamical tracking of human respiration, however, in the current version of the prototype system, only one electrode can be used with a sampling rate *1/16*. Following versions of the prototype will need to address this issue in order to be able to measure all eight channels with higher frequency sampling rates.

## 4. Conclusions

A novel prototype QCM sensor array for human breath/respiratory analysis was fabricated, based on the preliminary home-made experimental system reported in a previous publication [22]. Device operation parameters (temperature, flow, and frequency sampling rate) were optimized for human respiratory signal acquisition. Humidity responses and respiratory signals at different temperatures were investigated for the use of QCM electrodes with different types of coatings. Due to the possible non-linear sensor responses at high humidities, and based on the speed of response, a device operating at a temperature equal to 35 °C was selected as optimal. Optimal frequency sampling rates of, at least 1/8, were found to be essential for a detailed respiratory pattern record, which is needed for lung function tests; however, sampling rates up to 1/1 are sufficient for respiratory rate measurement, and this result confirms the conclusions of the first report. With the extendeded capability of the system to hold and simultaneously use up to eight electrodes, future experiments will be designed to realize the detection of certain chemical compounds in breath, simultaneously, with lung function tests. A combination of eight electrodes with different functional coatings, in principle, should allow the creation of a QCM-based spirometer and electronic nose in one device platform. We have demonstrated that QCM sensors modified with porous SiO_2_ NPs with infused receptors (TSPP, SCA-8, β-SCD) show higher responses to the normal respiration, compared to three types of flat nano-thin LbL films.

To the best of our knowledge, the developed prototype system is the first QCM-based machine for human respiration assessment. A larger field trial of the described prototype would be the best justification of its analytical value, however, it requires the involvement of humans and, thus, has relevant ethical issues. We believe that extensive laboratory tests for validation of the device using conventional techniques, involving small groups of healthy individuals, are an essential step before we move into field tests.
